# Solar radiation explains litter degradation along alpine elevation gradients better than other climatic or edaphic parameters

**DOI:** 10.3389/fmicb.2023.1152187

**Published:** 2023-04-27

**Authors:** Sarah Semeraro, Pascal Kipf, Renée-Claire Le Bayon, Sergio Rasmann

**Affiliations:** Laboratory of Functional Ecology, Institute of Biology, University of Neuchâtel, Neuchâtel, Switzerland

**Keywords:** soil physicochemical properties, alpine soils, elevation gradients, soil microbial respiration, tea bag experiment

## Abstract

Organic matter (OM) decomposition has been shown to vary across ecosystems, suggesting that variation in local ecological conditions influences this process. A better understanding of the ecological factors driving OM decomposition rates will allow to better predict the effect of ecosystem changes on the carbon cycle. While temperature and humidity have been put forward as the main drivers of OM decomposition, the concomitant role of other ecosystem properties, such as soil physicochemical properties, and local microbial communities, remains to be investigated within large-scale ecological gradients. To address this gap, we measured the decomposition of a standardized OM source – green tea and rooibos tea – across 24 sites spread within a full factorial design including elevation and exposition, and across two distinct bioclimatic regions in the Swiss Alps. By analyzing OM decomposition via 19 climatic, edaphic or soil microbial activity-related variables, which strongly varied across sites, we identified solar radiation as the primary source of variation of both green and rooibos teabags decomposition rate. This study thus highlights that while most variables, such as temperature or humidity, as well as soil microbial activity, do impact decomposition process, in combination with the measured pedo-climatic niche, solar radiation, very likely by means of indirect effects, best captures variation in OM degradation. For instance, high solar radiation might favor photodegradation, in turn speeding up the decomposition activity of the local microbial communities. Future work should thus disentangle the synergistic effects of the unique local microbial community and solar radiation on OM decomposition across different habitats.

## 1. Introduction

The global carbon cycle is at the base of the functioning of most ecosystems (Schimel, 1995; Stuart Chapin et al., 2009; Shukla et al., 2019) and is primarily driven by climatic (temperature and precipitation) factors (Bardgett et al., 2008; Lal, 2013; Wagg et al., 2021; Jungkunst et al., 2022). Because climate change is accelerating, understanding climate’s effects on the carbon cycle is of primordial importance (Davidson and Janssens, 2006). Among many, one of the critical events driving the carbon cycle, is the rate at which organic matter (OM) decomposes once it reaches the topsoils’ layers. Indeed, OM added to the soils can take two main pathways: incorporation into soil organic layers or mineralization (Angst et al., 2021). In the first pathway, OM may persist in soils, sometimes for thousands of years (Schmidt et al., 2011), where it will be integrated into the organo-mineral layers, stocking up the soil’s organic carbon bank (Man et al., 2022). In the second pathway, the decay of dead plant and animal material (i.e., litter) transforms complex organic molecules into simpler organic and inorganic molecules. Then, via the consumption by detritivores, litter is transformed into energy, and CO_2_ is released back into the atmosphere. Therefore, OM decomposition fuels energy flow within the soil biota, i.e., the macro- and mesofauna, but especially the microbial (bacterial and fungal) communities (Krishna and Mohan, 2017).

Organic matter decomposition is influenced by various biotic and abiotic factors (Bhatnagar et al., 2018; Woolf and Lehmann, 2019). For instance, plant litter quality has been shown to influence decomposition rates, in which more recalcitrant molecules should slow down the decomposition process, whereas litter containing high nitrogen to lignin ratio should accelerate it (Murphy et al., 1998; Cornwell et al., 2008; Berg, 2014; Chomel et al., 2016; Lu et al., 2017; Djukic et al., 2018). Moreover, other facets of the ecosystems have been shown to also affect OM decomposition across habitats (Schmidt et al., 2011), including soil physicochemical properties (Liu et al., 2006; Ge et al., 2013; Delgado-Baquerizo et al., 2015), temperature and precipitations (Meentemeyer, 1978; Aerts, 1997; Fierer et al., 2005; Aerts, 2006) and soil communities’ (i.e., decomposers) activity (Bray et al., 2012; Tresch et al., 2019; Sokol et al., 2022). In addition, while each variable can affect OM decomposition independently, Bradford et al. (2016) proposed to also consider the concomitant action of multiple ecological factors simultaneously. Therefore, predicting the relative influence of the different components of the ecosystem on OM decomposition in the soils remains a hard nut to crack (Carrenho et al., 2020).

In this context, large-scale ecological gradients can be used to tease apart the effect of different ecological factors on OM decomposition (Krishna and Mohan, 2017). For instance, decomposition rates have been shown to decrease with latitude (Zhang et al., 2008) and elevation (Sundqvist et al., 2011), likely due to a decrease in temperature and time of the growing season. However, along ecological gradients, multiple variables co-vary with climate, making it difficult to disentangle the contribution of each variable (Zhu et al., 2019). For instance, plant community beta diversity - and the inherent phytochemical composition - co-vary with other variables such as temperature and humidity (Defossez et al., 2021). To resolve this conundrum, one way is to harmonize leaf litter decomposition studies by using a common source of litter for measuring OM decomposition across different ecosystems. In this regard, Keuskamp et al. (2013) developed the tea bag method, using standardized tea types (green tea and rooibos tea as substrate) to explore the effect of local factors on litter decomposition, independently of the site-specific vegetation differences in phytochemistry (Defossez et al., 2021). For example, Didion et al. (2016) contrasted teabags’ degradation rates and decomposition of local litterbags along an elevation gradient. They observed the slowest decomposition rates at the high-elevation site, independently of the litter quality. Therefore, elevation alone bears poor explanatory power for studying variation in OM decomposition. However, by studying OM decomposition across multiple orthogonal axes, in conjunction with a standardized litter decomposition assay, might help tease apart the relative importance of the different ecological factors, such as climatic and soil factors, driving variation in OM decomposition.

Therefore, to further shed light on the relative contribution of different ecological factors (biotic and abiotic parameters, see Supplementary Table S2) driving OM decomposition across habitats, we took advantage of the natural climatic and edaphic variation along the steep elevation gradients of the Alps (Hagedorn et al., 2019). Specifically, we asked which edaphic, climatic, or microbial activity-related variables, best explain OM decomposition rates across sites. To address this question, we measured litter decomposition using the teabag approach (Keuskamp et al., 2013). We assessed the effect of different climatic and edaphic variables across two regions (northern Prealps versus southern Alps), two elevation zones (alpine versus subalpine), and two expositions (north versus south). Specifically, we hypothesized that by generating variation in ecological variables potentially driving OM decomposition using a common litter source, it would be possible to identify key variables best-explaining variation in OM decomposition. Ultimately, by dissecting the relative role of biotic and abiotic factors driving soil OM degradation, this work should provide additional information on how to estimate the rate of carbon turnover across ecosystems, and the effect of climate change key natural ecosystem processes.

## 2. Materials and methods

### 2.1. Study sites and climatic variables

**Figure 1:**
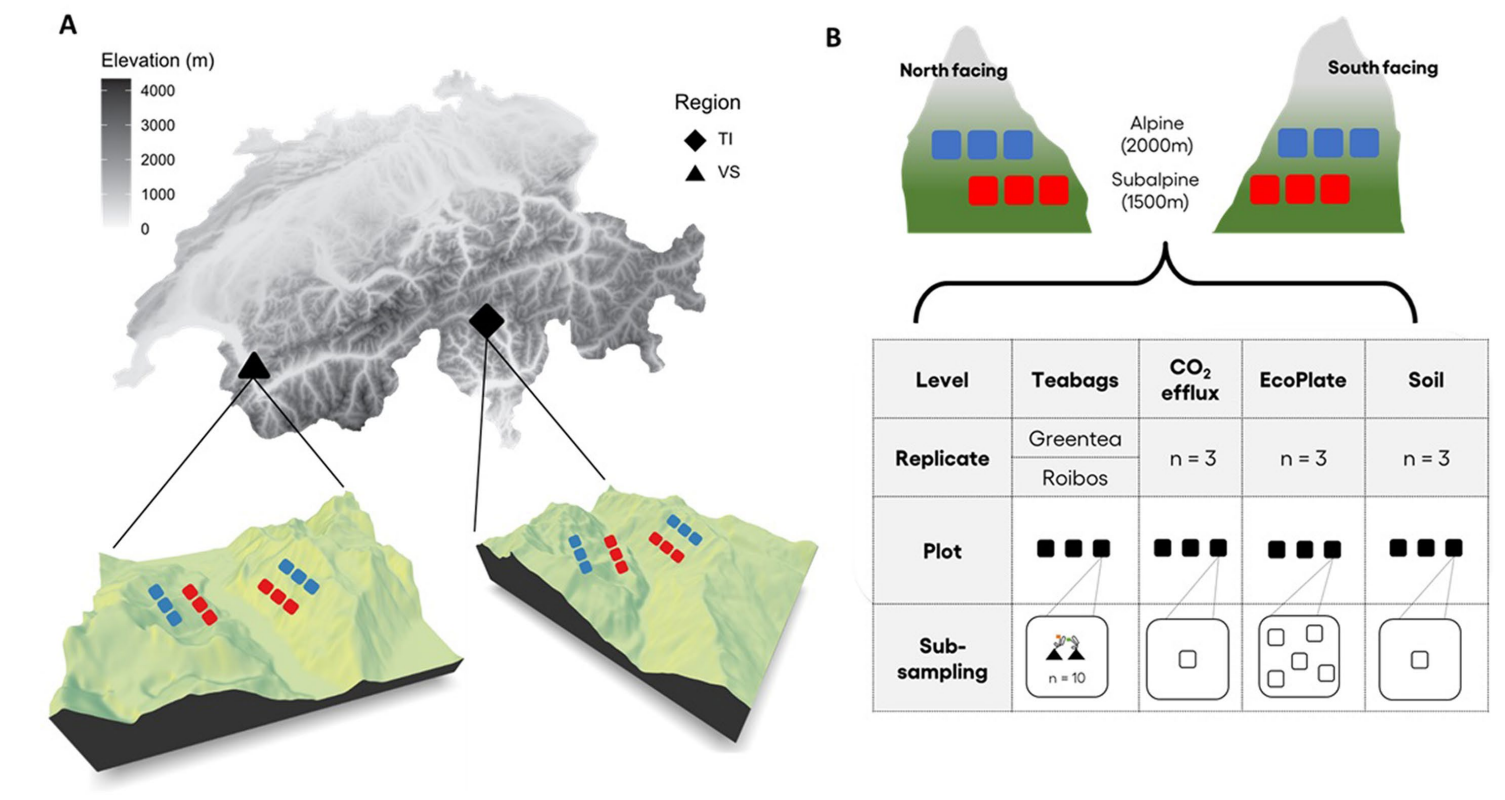
Study sites and experimental design. **(A)** Map of Switzerland showing the location of the two regions where the study took place; Valais (*VS*, black triangle) transect in the Northern Alps, and Ticino (TI, black diamond) in the Southern Alps. In both regions, we established a north- and a south-facing altitudinal transect. **(B)** The elevation transects were divided into two elevation zones; a subalpine zone (1,500 m a.s.l, red squares), and an alpine zone (2,100 m a.s.l., blue squares). On each transect and at each elevation, we defined three replicate plots (*N* = 2 regions × 2 expositions × 2 elevation zones × 3 replicates = 24 plots). The graphic shows the different variables sampled in each plot: (i) soil profile description (*n* = 1) and soil physicochemical parameters therein, (ii) soil respiration (CO_2_ efflux) measures (*n* = 3), (iii) carbon degradation bioassay (EcoPlates, *n* = 5), (iv) tea bags experiment (*n* = 10 per plot and per tea type: green tea and rooibos tea).

To identify the climatic and edaphic drivers of leaf OM decomposition, we performed a litter bag experiment using two standardized tea types [Rooibos and Green tea (Keuskamp et al., 2013)] across 24 sites in the Alps. The experiment was replicated across two distinct bioclimatic regions (the Northern Prealps in the canton of Valais in Switzerland, and the Southern Alps, in the canton of Ticino in Switzerland, Figure 1). In each region, we selected two elevation transects facing each other, a north and a south-facing slope. Within each elevation transect, we randomly selected three plots, at the subalpine level [approximately 1,500 m above sea level (asl)] and the alpine level (about 2,100 m asl). Each plot was distant from the other by at least 100 m (N = 2 regions × 2 expositions × 2 elevations × 3 replicates = 24 plots). Plot size varied according to the vegetation type; at the subalpine level, dominated by conifer forests, the vegetation plots were 400 m^2^ (20 m × 20 m), whereas, at the alpine level, dominated by alpine grasslands, the vegetation plots were 25 m^2^ (5 m × 5 m). To assess the site-specific climate, for each site, we extracted environmental data, including elevation, number of degree-days, solar radiation, number of frost days, and potential evapotranspiration (hereafter referred to as moisture) from associated environmental layers (Pellissier et al., 2016). We calculated values for temperature (degree-days) and moisture from meteorological stations using a Digital Elevation Model (DEM) at 25 m resolution and interpolated the following (Zimmermann and Kienast, 1999). We estimated solar radiation values using the tool implemented in ArcGIS 10 (Zimmermann and Kienast, 1999).

### 2.2. Soil sampling and analysis

To estimate the effect of soil physicochemical parameters on litter decomposition, we analyzed physicochemical properties of the topsoils (A horizons) for each plot, since the OM content and turnover are highest in this layer. Specifically, for each plot, 5–10 soil samples were randomly collected with a soil borer (5 cm in diameter, 10–20 cm deep), homogenized, dried at 40°C, and then sieved at 2 mm. We measured six variables: (1) pH, measured in distilled water with a Metrohm 827 pH meter (Metrohm AG, Herisa, Switzerland). (2) Soil OM, measured from the loss of ignition (Allen et al., 1974) and corrected by the ‘Howard’ correction factor (Howard and Howard, 1990). (3) Total cationic exchange capacity (CEC), determined using the ‘cobalt hexamine trichloride’ method (Ciesielski et al., 1997). (4) Total carbonates, quantified by CaCO_3_ decomposition after adding HCl in CO_2_ and water using the Calcimeter Bernard method (Allison and Moodie, 1965). (5) Total carbon (C) and nitrogen (N) were quantified using an elemental analyzer (FLASH 2000, Thermo Scientific, Waltham, Massachusetts, United States). (6). Soil relative humidity (Rh) was assessed after drying the soils at 105°C for 3 days.

### 2.3. Tea bag experiment

To determine the site-specific decomposition rate of organic material, we buried two different types of tea bags following standard procedures (Keuskamp et al., 2013). Burial took place during September and October 2020, until collection in May and June 2021 (see Supplementary Table S3 for precise dates of burial time). On each site, Lipton Green Tea Sencha (European Article Number (EAN No): 8714100 77,054 2, *n* = 15) and Lipton Rooibos Tea (EAN No.: 8722700 18,843 8, *n* = 15) were buried in pairwise fashion (one Green Tea and one Rooibos bag together) with 5–10 cm to each other at a soil depth of 5 cm within 15 m to the right of the plot center (Figure 1B). The burial locations were chosen as to be representative of the general vegetation of the experimental plot. After 8–9 months, tea bags were carefully excavated, wiped off adherent soil particles, and dried in paper envelopes at 40°C for at least 48 h. The decomposition rate (k – percent of weight loss per day) for both tea types was next assessed using initial weight (w_i_), final weight (w_f_), and burial time as follows:


$$k=\frac{\left(\frac{w_{i}-w_{f}}{w_{f}\times 100}\right)}{\;\mathrm{Burial}\;\mathrm{time}}$$

### 2.4. Soil microbial carbon degradation bioassay

To determine the potential of the local soil microbial community to degrade OM, we used a BIOLOG EcoPlate™ bioassay (Grządziel and Gałązka, 2018). For this, in each plot, five randomly chosen soil subsamples (4 cm diameter × 20 cm deep) were homogenized in a plastic container and stored at 5°C in the lab for a maximum of 24 h. One gram of soil per sample was then added with 99 ml of 0.9% saline solution (NaCl), agitated at 145 rpm for 30 min at room temperature, again cooled at 4°C for 30 min, and vortexed for 15 s before pouring the supernatant (microbial suspension) into a sterile petri dish. Next, 120 μl of each microbial suspension was added to each cell of the EcoPlate. Each plate comprises 96 wells with 32 carbon sources (including blank) carbon sources in triplicates (Supplementary Figure S1). Photometric readings (λ = 590 nm) were carried out using the Biochrom Asys UVM 340 Scanning Microplate Reader and the ASYS DigiRead software. Three measurements in immediate succession were done in the morning and the evening for 8 days after the analysis started. The higher the optical density reading the more the carbon source has been used. For our study, we first analyzed the temporal dynamics of total carbon consumption in each plate (i.e., the sum of each carbon source consumed, Supplementary Figure S2), and chose the average of the two readings at day five as the maximal carbon source consumption for response variable to compare across sites.

### 2.5. Soil respiration

To obtain an indirect measure of potential soil biological activity (including microbial, faunal and root activity) at each site, we performed soil respiration measurements (CO_2_ effluxes, *n* = 72) with a portable LI-8100A Automated Soil Gas Flow System (LI-COR Biosciences GmbH, Bad Homburg, Germany). In each plot, we randomly chose three measurement spots (Figure 1B) where there was bare soil without vegetation cover in forests, and where vegetation cover was representative for the rest of the plot’s vegetation in the alpine zone, avoiding shrubby vegetation or bare soil. Before measurements, we hammered a PVC collar (20 cm Ø chamber) into the soil perpendicularly to the slope. Where the soil was too compacted or with many roots, insertion was facilitated by cutting along the collar with a pocketknife. Under sunny conditions, the setup was shaded by an umbrella to protect the detection unit from possible interference by direct UV radiation. Measurements were taken for 2 min and repeated twice (Supplementary Table S4). We took as response variable the average of the intercept of the liner regression of the two measurements as provided by the LI-COR software.

### 2.6. Statistical analysis

All analyses were performed in R 4.1.1 (R Development Core Team, 2020).

**Figure 2:**
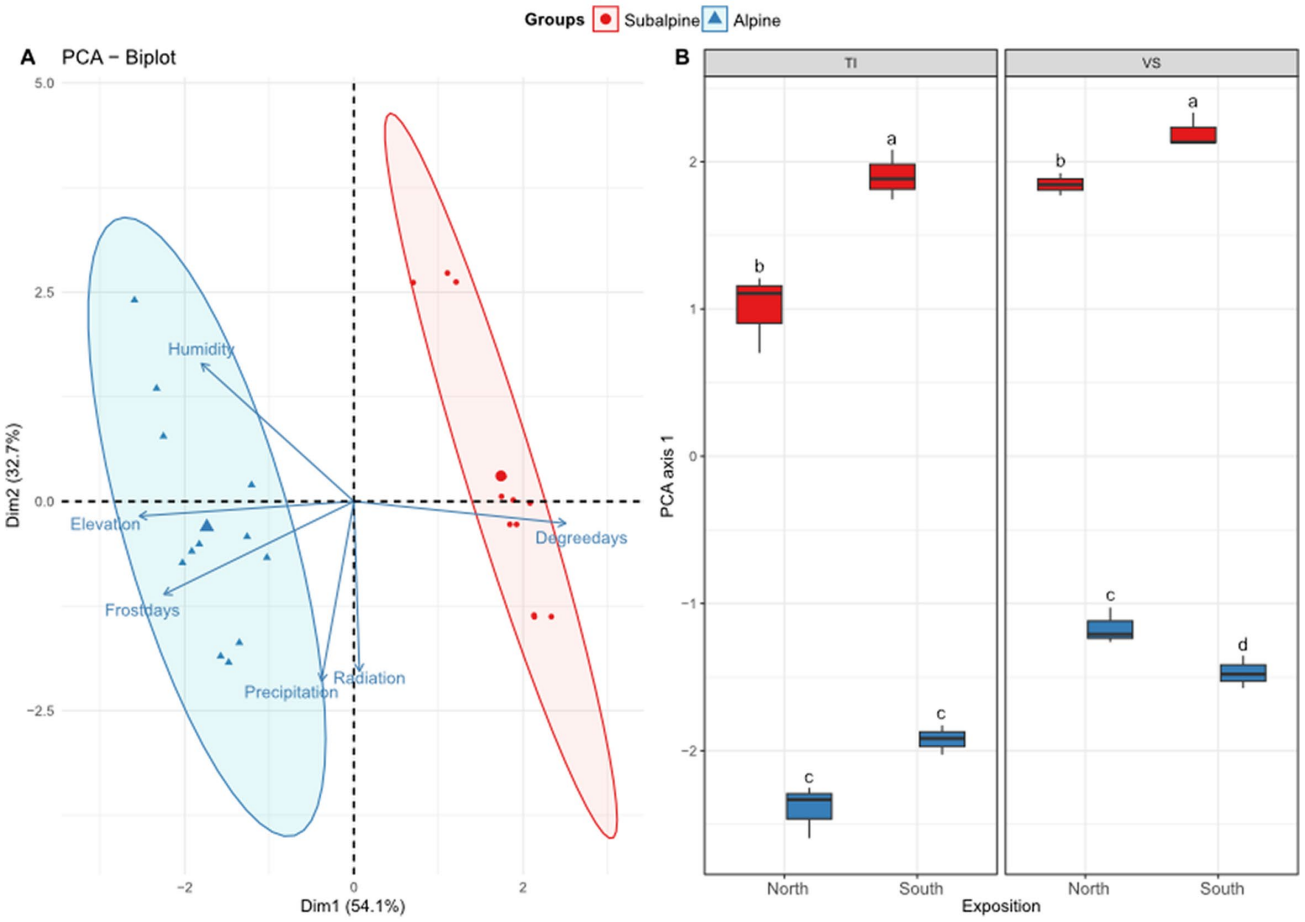
Climatic niche. Shown is **(A)** the principal component analysis (PCA) biplot of six climatic variables retained in the analysis: relative evapotranspiration (humidity), elevation of each site, number of frost (<0°C) days during the year (frost days), annual mean precipitation (precipitation), annual mean solar radiation (radiation), average number of degree-days per year (degree days). **(B)** Boxplots representing average values along the first axis of the PCA separated by exposition (north versus south facing slopes), and regions (Ticino = TI, or Valais = *VS*). Letters above boxplots represent significant differences among main effects (Tuckey’s post-hoc tests; *p* < 0.05). Red color (warm) represents the subalpine zone (~1,400 m above sea level), and blue color (cold) represents the alpine zone (~2000 m above sea level).

*Climatic variables* – We visualized correlations among the climatic variables across sites using principal component analysis (PCA), calculated using the ade4 package (Dray and Dufour, 2007). We assessed the interactive effect of elevation zone (subalpine and alpine) and exposition (north and south) using a Regularized Discriminant Analysis (RDA; Friedman, 1989) using the vegan package (Oksanen et al., 2013). We included the region as a blocking factor in the analysis. In addition, to estimate multivariate changes of the climatic conditions across sites (see Figure 2A), we performed a two-way ANOVA (zone * exposition + region) on the first axis of the PCA (PCA1 in Figure 2A).

**Figure 3:**
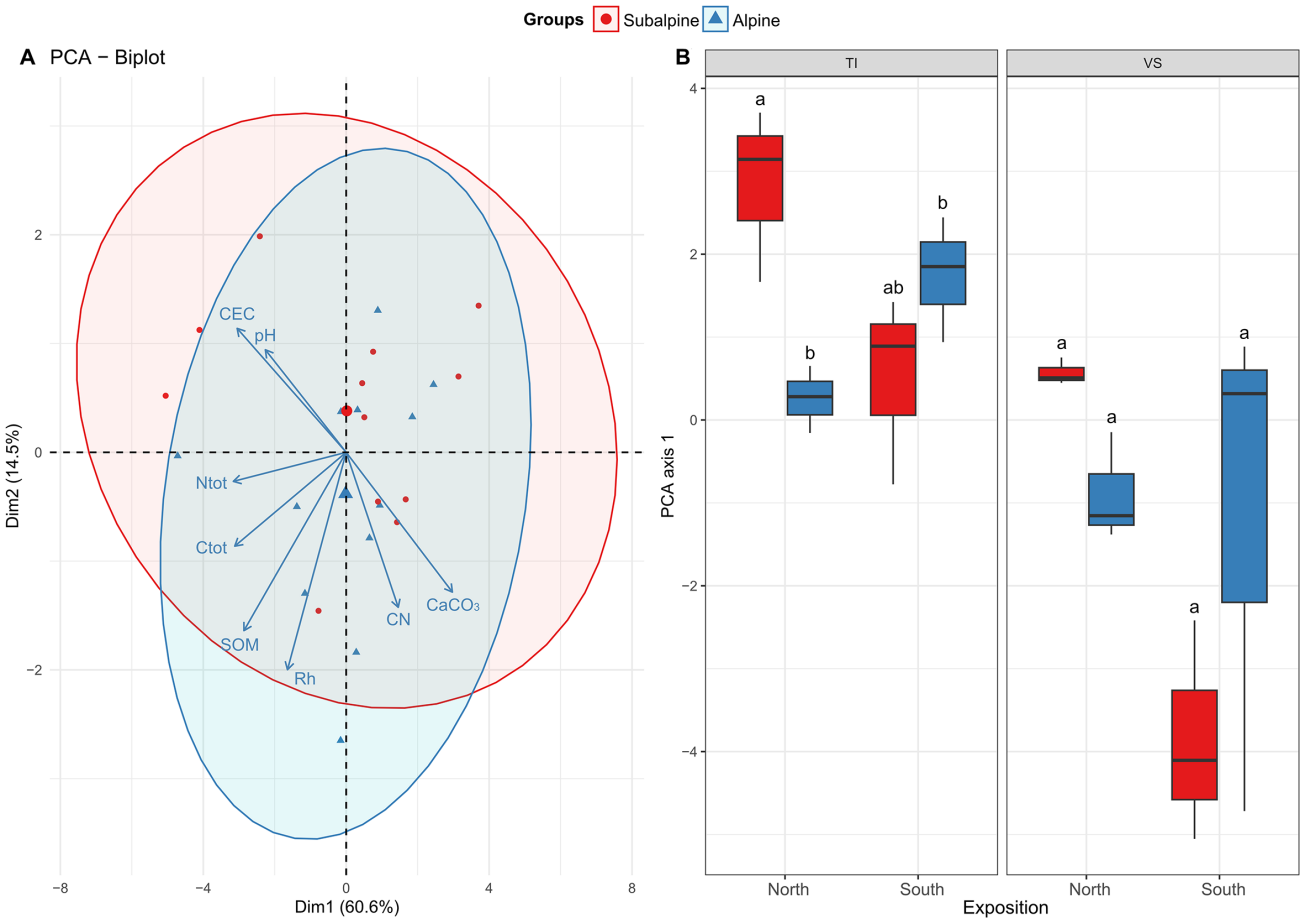
Edaphic properties. Shown is **(A)** the principal component analysis (PCA) biplot of eight physicochemical soil variables [pH, Cation-Exchange Capacity (CEC), total nitrogen (N_tot_), total carbon (C_tot_), soil OM (SOM), soil relative humidity (Rh), carbon-to-nitrogen ratio (C/N), and total carbonates (CaCO_3_)]. **(B)** Boxplots representing average values along the first axis of the PCA separated by exposition (North versus South facing slopes), and regions (Ticino = TI, or Valais = *VS*). Letters above boxplots represent significant differences among main effects (Tuckey’s post-hoc tests; *p* < 0.05). Red color (warm) represents the subalpine zone (~1,400 m above sea level), and blue color (cold) represents the alpine zone (~2000 m above sea level).

*Soil physicochemical properties* – We visualized differences in the physicochemical properties of soils using principal component analysis (PCA) as described above. We assessed the effect of elevation zone on soil physicochemical properties using a Regularized Discriminant Analysis (RDA; Friedman, 1989). We included the region as a blocking factor in the analysis. To assess how the soils’ multivariate space changes along the elevation gradient, we performed a two-way ANOVA (zone*exposition + region) on the first axis of the PCA (see Figure 3A).

*Tea bags degradation, soil microbial carbon degradation and soil respiration* – To analyze site differences for tea bag degradation, soil carbon degradation and soil respiration, we performed two-way ANOVAs, separately for each response variable, with elevation zone and exposition as fixed factors, and the region as blocking factor.

*The relative importance of the measured variables for tea bags degradation* – To finally assess the relative importance of the different explanatory variables (edaphic, climatic, soil respiration and microbial carbon degradation) on tea litter decomposition, we performed a Random Forest Regression with the rfPermute package in R (Archer and Archer, 2016), which allows estimating the significance of the importance of the variables for a Random Forest model by permuting the response variables and producing a null distribution of important metrics for each predictor variable. Based on analysis of the random forest analysis results, we next performed univariate linear regressions between the explanatory variables deemed significant (see Supplementary Figures S3, S4) and the k values for green tea and rooibos tea.

## 3. Results

### 3.1. Climatic variables

Based on the ordination analyses, we confirmed that alpine zones display colder (7 times more frost days, and 40% less degree-days) and more humid conditions (6% more annual average precipitation days, and 2.3 times more humidity values) than subalpine zones (Figure 2A; RDA analysis for elevation zone effect; *F*_1,19_ = 39.01, *p* < 0.001). North facing slopes were also colder and more humid (8% more degree-days, 26% more solar radiation, 50% less humidity, 7% more precipitations, 26% more frost days) than south-facing slopes (exposition effect; *F*_1,19_ = 17.99, *p* < 0.001). We also found a significant effect of the region (*F*_1,19_ = 18.21, *p* < 0.001), in which the Valais (*VS*) region displayed 3% more solar radiation, 17% more precipitation and 2.64 times less overall humidity than the Ticino (TI) region (Figure 2B). However, the interaction between elevation and exposition was not significant (*F*_1,19_ = 0.96, *p* = 0.382), suggesting that the climatic patterns of warmer and drier conditions on the south-facing slopes were consistent across elevation zones (Figure 1B; Table 1).

**Table 1:** Two-way ANOVA table for the effect of elevation zone (two levels; alpine and subalpine), exposition (two levels; north and south), region (two levels; Valais, Ticino), and interaction between zone and exposition.

Variable	Factor	Df	SSQ	*F* value	Pr (<*F*)	
PCA1 - clim	Elevation Zone (Z)	1	72.51	1187.931	<2e–16	***
	Exposition (E)	1	0.75	12.320	0.00234	**
	Region	1	2.97	48.713	1.19e-06	***
	Z * E	1	0.44	7.242	0.01446	*
	Residuals	19	1.16			
PCA1 – soil	Elevation Zone (Z)	1	0.01	0.003	0.957297	
	Exposition (E)	1	11.53	5.776	0.026617	*
	Region	1	43.01	21.537	0.000178	***
	Z * E	1	23.79	11.911	0.002675	**
	Residuals	19	37.94			
Soil respiration	Elevation Zone (Z)	1	3.838	45.257	1.98e–06	***
	Exposition (E)	1	0.004	0.049	0.826	
	Region	1	0.000	0.003	0.957	
	Z * E	1	0.102	1.199	0.287	
	Residuals	19	1.611			
C degradation	Elevation Zone (Z)	1	499.0	24.324	9.26e–05	***
	Exposition (E)	1	47.1	2.298	0.146018	
	Region	1	354.5	17.280	0.000535	***
	Z * E	1	10.2	0.498	0.489048	
	Residuals	19	389.7			
Tea bag decomposition	Elevation Zone (Z)	1	0.00895	13.151	0.00036	***
	Exposition (E)	1	0.04541	66.762	2.75e–14	***
	Region	1	0.00008	0.114	0.73646	
	Z * E	1	0.00215	3.157	0.07705	.
	Residuals	212	0.14420			

Significance codes: 0 ‘***’ 0.001 ‘**’ 0.01 ‘*’ 0.05 ‘.’ 0.1 ‘ ’ 1.

### 3.2. Edaphic variables

The ordination analysis highlights a non-significant effect of the elevation on soil variables, indicating that soil physico-chemical properties, overall, were rather similar across the alpine and the subalpine. Yet, we found a significant effect region and exposition (Figure 3A; Table 1), meaning our soils across our 24 sites were overall quite different (Figure 3B, and see details Supplementary Figure S5, Supplementary Table S1). We found that *VS* soils contained 1.92 times more N than the TI soils. Moreover, the total N content increases from north to south in the subalpine zone. In contrast, in the alpine zone, we found the reversed trend (see the interaction between zone and exposition in Supplementary Table S1), a trend that was mirrored for the total carbon content (C_tot_). For carbon-to-nitrogen ratio (C/N), it was 64% higher in the TI region than in the *VS* region, while the residual soil humidity (Rh) was only significantly higher in the alpine soils versus the subalpine soils. Moreover, *VS* soils were 2.6 times more alkaline than the TI soils, while *VS* soils were 1.24 times richer in OM than the TI soils. For soil cation-exchange-capacity (CEC), we found an effect of the elevation and exposition, in which subalpine soils were 1.70 times higher in CEC than alpine soils, and south-exposed soils were 2.58 higher in CEC than north-exposed soil. Also, CEC for *VS* was 3.96 times higher than in TI. For soil total carbonate content (CaCO_3_), we found an effect of the elevation and exposition, in which alpine soils were 1.17 times more calcareous than subalpine soils, and north-exposed soils were 1.29 times more calcareous than south-exposed soils. We also found that TI soils were 1.42 times more calcareous than *VS* soils, and that carbonate content decreased from north to south in the subalpine zone. In contrast, in the alpine zone, carbonate remained similar (see the interaction between zone and exposition in Supplementary Table S1). Concerning the soil mineralogy, we found, that *VS* soils contained 3.66 times more clays and 1.81 more silt than the TI soils. However, TI soils were 3.08 times more sand-rich than VSs soils. Alpine soils were 1.23 times sandier than subalpine soils, and north-exposed soils were 1.28 times sandier than south-exposed soils (Supplementary Table S1).

### 3.3. Soil respiration, carbon degradation, and tea litter decomposition

**Figure 4:**
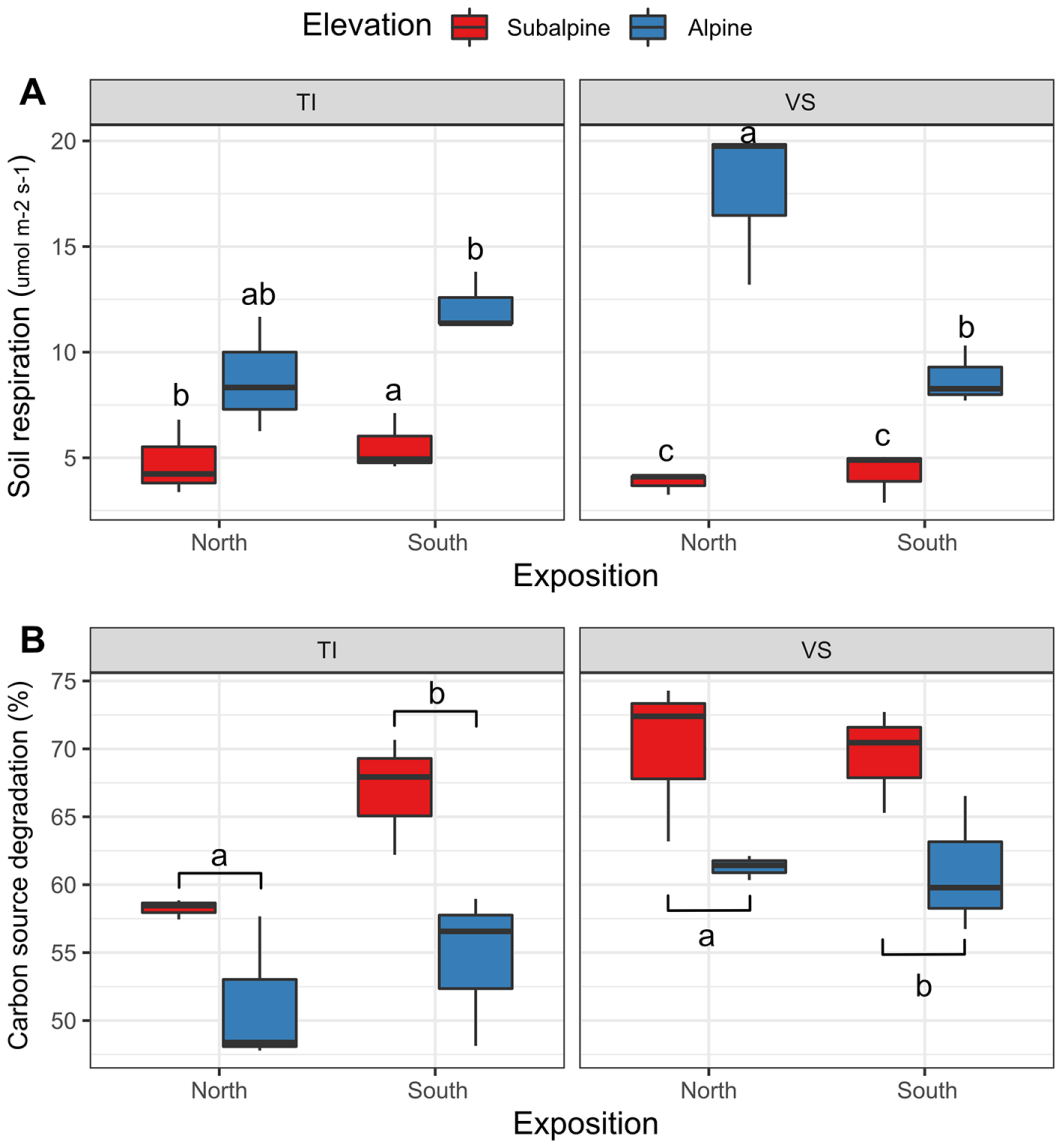
Soil respiration and microbial activity. Shown is **(A)** Boxplots representing average soil respiration (Licor bioassay), and **(B)** average soil carbon degradation (EcoPlates bioassay) values at each site separated by exposition (North versus South facing slopes), and regions (Ticino = TI, or Valais = *VS*). Letters above boxplots represent significant differences among main effects (Tuckey’s post-hoc tests; *p* < 0.05). Red color (warm) represents the sub-alpine zone (~1,400 m above sea level), and blue color (cold) represents the alpine zone (~2000 m above sea level). *N* = 3 plots per site.

We found that alpine soils respired 2.56 times more than subalpine soils (Figure 4A, see elevation effect in Table 1). However, we found a non-significant effect for exposition, region, and elevation by exposition (Table 1). Contrary to soil respiration, the microbial carbon consumption was 16% higher in the subalpine than in the alpine elevation zones (Figure 4B; Table 1), independently of the exposition (see the non-significant effect of exposition and exposition by elevation interaction in Table 1). Moreover, soils from the Valais region, in the northern part of the Alps, contained 13% more active microbial communities than soils from south of the Alps.

**Figure 5:**
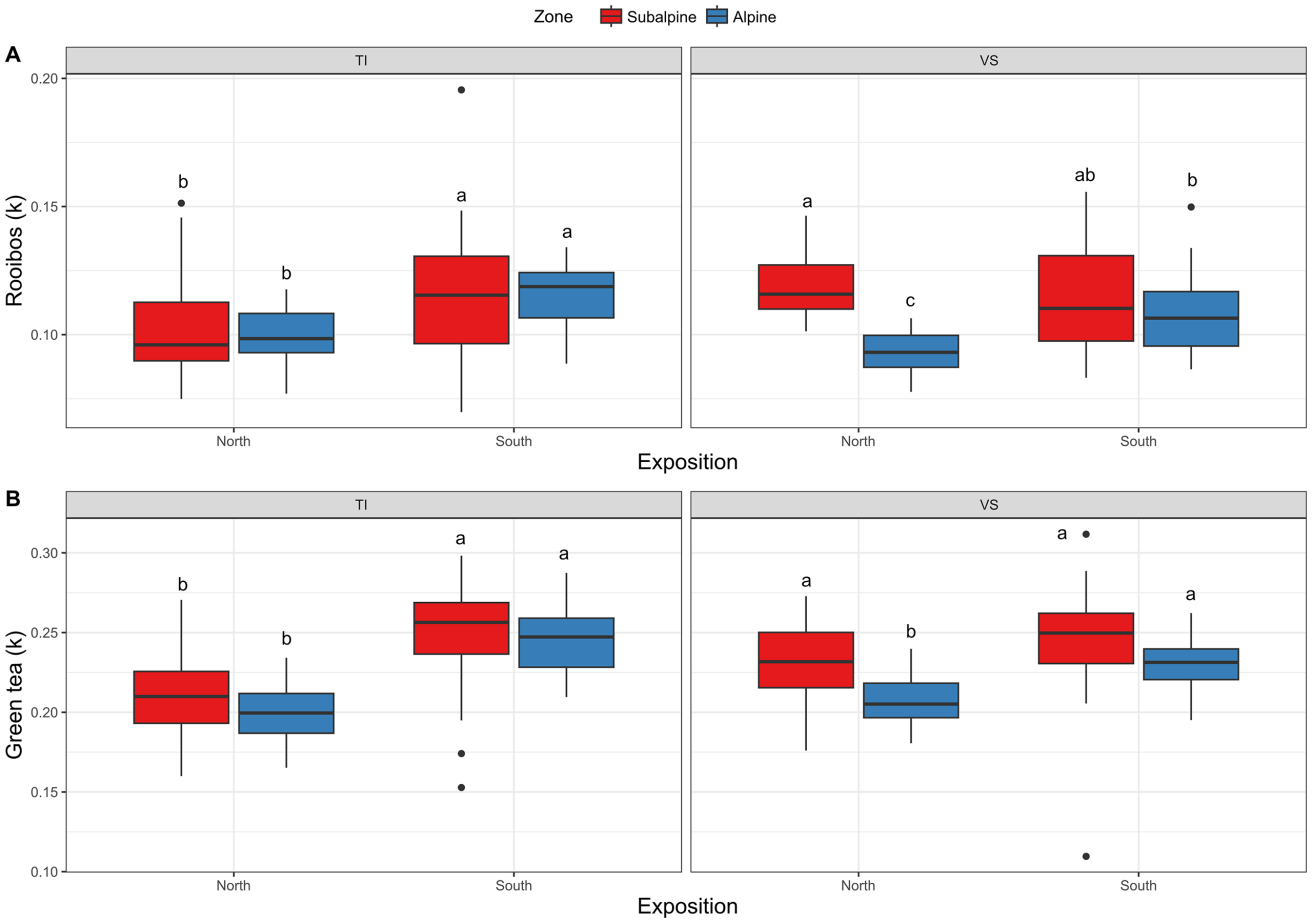
Degradation of teabags. Shown is **(A)** Boxplots representing rooibos degradation (k values), and **(B)** green tea degradation (k values) at each site separated by exposition (North versus South facing slopes), and regions (Ticino = TI, or Valais = *VS*). Red color (warm) represents the sub-alpine zone (~1,400 m above sea level), and blue color (cold) represents the alpine zone (~2000 m above sea level). *N* = 3 plots per site. The *k* values represent the percent of tea weight loss per day.

Concerning the teabags bioassays, overall green tea degraded slightly faster in the subalpine (*k* = 0.23) than in the alpine (*k* = 0.22; Figure 5A; Table 1) and 5% more on south-facing than on north-facing slopes (Figure 5A; Table 1). These effects were similar for the rooibos tea bags, degrading 8% faster on the subalpine than on the alpine elevation zones (Figure 5B; Table 1), and 5% faster on south-facing than on north-facing slopes (Figure 5B; Table 1).

### 3.4. The relative importance of the predictor variables

**Figure 6:**
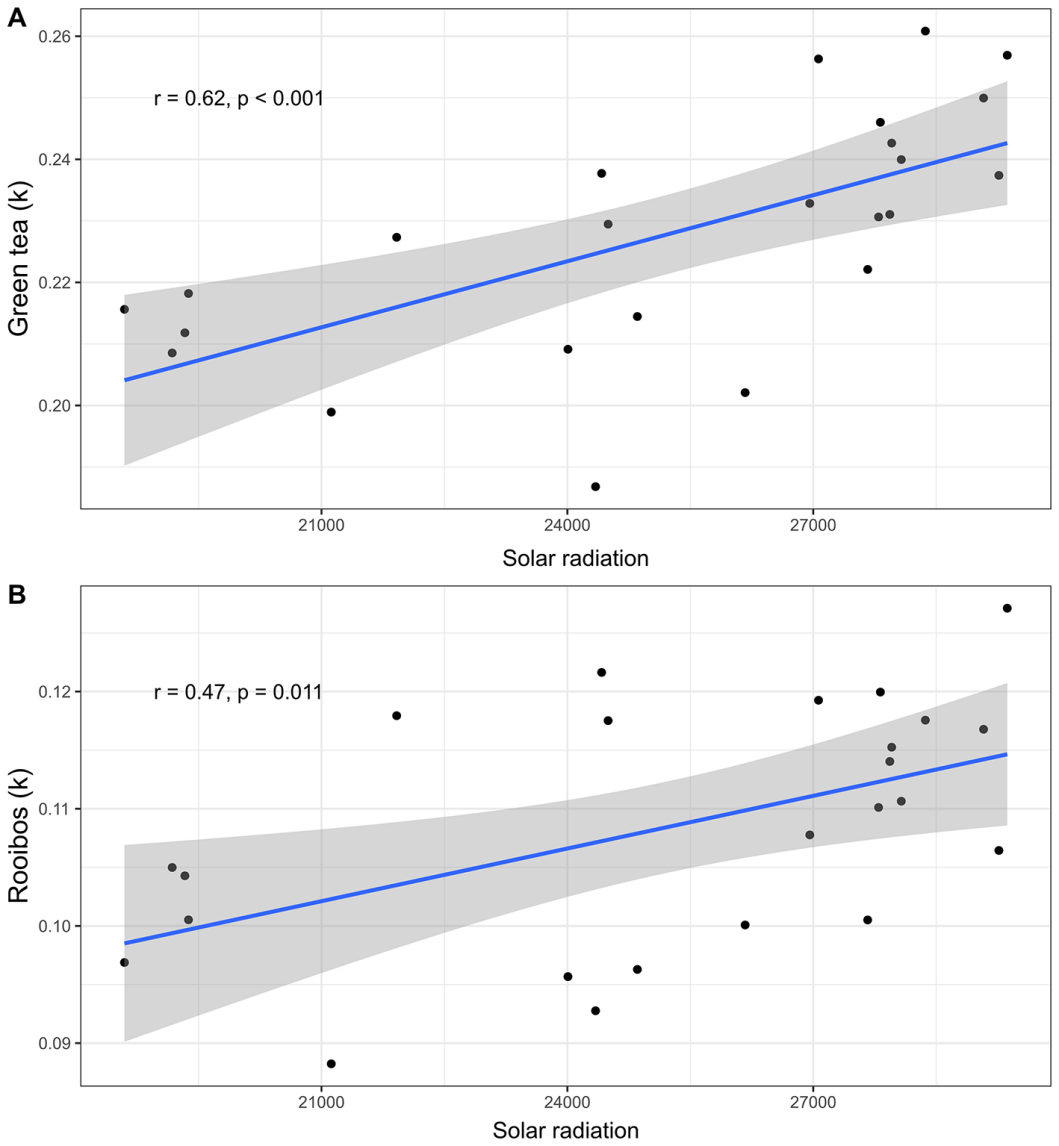
Effect of solar radiation on teabags decomposition. Scatterplots displaying liner regressions (blue lines with confidence intervals in grey) between solar radiation and k values for **(A)** green tea (linear regression; *n* = 24, r = 0.62, *p* < 0.001), and **(B)** rooibos tea (linear regression; *n* = 24, r = 0.47, *p* = 0.011).

The Random Forest Analysis indicated that solar radiation best explained degradation across sites for both the green tea and rooibos tea (Supplementary Figures S3, S4, respectively). The higher the average annual solar radiation the site received, the more likely the litter falling on the ground was to be degraded (for green tea, Figure 6A, lm; *F*_1,22_ = 15.98, *p* < 0.001, and for rooibos tea, Figure 6B, *F*_1,22_ = 7.65, *p* = 0.01). Moreover, the humidity was the second-best predictor for both tea types, followed by soil clay content (Supplementary Figures S3, S4).

## 4. Discussion

We studied the decomposition of two common litter types (green tea and rooibos tea) across 24 sites in the Alps, focusing on climatic, physicochemical soil properties, and variables related to soil microbial activity (19 in total). We found that solar radiation best captured the variation in litter degradation more so than other climatic or edaphic variables, including soil microbial activity. Below, we expand on the potential causes and consequences of the different variables affecting plant litter decomposition along large-scale ecological gradients.

### 4.1. The effect of climate on litter decomposition

The most stunning result of our study was to show that above all explanatory variables, variation in solar radiation across sites best-captured variation in tea bag degradation. These results align with multiple studies showing a positive effect of light (i.e., solar radiation) on photodegradation of litter (Hussain et al., 2023). For instance, it was shown that lower levels of UV-radiations inhibited litter decomposition in a Tibetan alpine steppe ecosystem (Mao et al., 2022), or that aboveground litter decomposition was predominantly controlled by photodegradation instead of litter phytochemical composition (Austin and Vivanco, 2006; King et al., 2012). Nonetheless, Hussain et al. (2023) in their review, point out that future research should focus on studying the interactions between different ecological factors, such as the potential interaction between photodegradation, soil moisture, and microbial communities. With this study, we contribute to filling this gap. That said, “more classic” climatic variables, such as mean annual temperature or precipitation (Carter, 2020), have also been shown to control the degree of soil OM decomposition along latitude (Wang et al., 2019) or elevation (Kong et al., 2022; Mao et al., 2022). Therefore, our results should not be taken as the fact that solar radiation, alone, controls all OM degradation in the Alps, but instead, that the variation we generated using our experimental design, generated higher variance in solar radiation, which ultimately best-explained litter degradation.

Indeed, as seen in the PCA of Figure 2, some climatic variables strongly co-varied with elevation, while others, such as solar radiation varied largely independently of elevation. Accordingly, we observed clear differences between subalpine and alpine climatic conditions across regions and south/north expositions. Generally, alpine and north-exposed sites display colder and more humid climate conditions, while subalpine and south-exposed environments displayed higher temperatures and dryer conditions, with higher levels of solar radiation. We found a non-significant interaction between the elevation zone and the exposition, indicating that warmer and drier climatic patterns were consistent across sites (Körner, 2007). Therefore, our experimental design generated variation in solar radiation, orthogonally to the elevation axis, and independently of the temperature/humidity axis (as shown in Figure 1B). Ultimately, this variation might have been enough to allow capturing most variation in k values for tea bags. Nonetheless, our experimental design might have induced the unintended effects of vegetation cover also influencing solar radiation reaching the ground, thus indirectly affecting our results. In other words, vegetation cover might have impacted the microclimatic understory conditions, hence also indirectly impacting OM degradation. Yet, we found that elevation zone *per se* did not impact the level of solar radiation (Supplementary Figure S6). In other words, we observed that the alpine and subalpine sites, overall, across the two regions received the same amount of solar radiation. Therefore, we can conclude that the observed effect of solar radiation on tea degradation was not or only partially driven by elevation differences and, therefore, not by vegetation cover differences, as the subalpine sites were always in forests, while alpine sites were always in grasslands. Perhaps, another way to understand these effects is to assume that solar radiation, here extrapolated from spatial models independent of vegetation cover, represents the amount of energy that a given ecosystem received. Hence, more energy in the system equates to faster metabolic activity and faster OM degradation (Caldwell et al., 1998).

Yet, according to classical theory and widespread ecological observations, alpine habitats are where degradation of OM is slowest, likely due to inhibitory effects of low average temperatures and short growing seasons (Hartley and Ineson, 2008; Wang et al., 2019). In our study we did not find this. Instead, tea leaves generally degraded similarly across elevation zones, suggesting that variation in temperature, and also perhaps humidity, was less important for OM degradation than other variables, in our case, solar radiation. However, it also may be that the difference of about 600 m between our subalpine and alpine sites (~3.6°C) was not sufficient to detect an effect of elevation *per se*, also due to differences in vegetation cover (see discussion above). In other words, differences in canopy cover between the subalpine forested areas and the alpine grasslands might have masked the temperature effect. Possibly, vegetation cover at the warmer subalpine level dampened high temperature on sunny days, while it increased the temperature effect disproportionally at high elevation on the same mountain slope, thereby homogenizing the real temperature effect at the soil level (i.e., for OM degradation). Future work would thus requires measuring microclimatic conditions for disentangling the canopy effect to average solar radiation effect at each site.

Even more so, such effect was independent of the type of tea, although, as predicted, rooibos tea degraded slower than green tea (Keuskamp et al., 2013). Yet, the mechanisms behind these effects driven by solar radiation remain to be fully elucidated. For instance, several studies argued that solar radiation can spur litter decomposition, by indirectly facilitating soil microbial decomposition via the weakening of the tissues through photodegradation (Lin et al., 2015; Marinho et al., 2020; Méndez et al., 2022). Moreover, we could speculate that within our study sites, higher solar radiation might correspond to ideal conditions of temperature and precipitation, in which OM degradation by the local soil fauna and flora is maximal. Further experiments are therefore required to test how the overall decomposition potential varies locally with increasing elevation and solar radiation. Manipulating solar radiations (i.e., photodegradation) at each site and over extended periods (which might trigger humidity and temperature changes) could confirm these hypotheses.

### 4.2. The effect of soil properties on litter decomposition

Through our experimental design, we also generated significant variations in soil physicochemical properties. For instance, we found major differences in primary soil mineral composition across Valais and Ticino soils, mainly due to extensive scale processes such as glaciation. Moreover, Valais soils (situated on a calcareous bedrock) and Ticino soils (situated on a crystalline bedrock) display similar soil types at the same elevations. However, their respective topsoil horizons differed in their primary composition, thus illustrating a distinct mineral composition between regions, which should directly affect soil carbonate content, and pH (Thomas and Hargrove, 1984). Furthermore, Valais soils were more fertile (based on CEC values) and alkaline (pH > 5), with more significant carbon and nitrogen contents than the Ticino soils.

Several soil properties have been shown to affect litter decomposition. For instance, parental material and soil mineral composition (i.e., calcareous or acidic soils) can affect litter and soil OM decomposition (Kooijman et al., 2005). This likely happens through parental material’s mediated changes in microbial and fungal community, and their inherent functional strategies for OM degradation (Komarova et al., 2022). Along these lines, soil texture and moisture have also been shown to alter litter decomposition (Angst et al., 2021; de Godoy Fernandes et al., 2021). High clay contents, for instance, play a significant role in soil fertility as major components of clay-humus complexes (Brydon and Sowden, 1959), providing more suitable conditions for soil microbial communities to use carbon from the litter. Indeed, clays and the clay-humus complex play an essential role in providing nutrients to the soil solution (Tahir and Marschner, 2017), affect soil water dynamics (Kumari and Mohan, 2021), and in turn, create favorable conditions for bacteria or fungi communities to develop (Högberg et al., 2013). In contrast, higher relative amounts of sand contribute to greater porosity and hydraulic conductivity within soils (Usowicz and Lipiec, 2021), thereby decreasing soil fertility and likely microbial activity for efficient litter decomposition.

In sum, while multiple soil parameters have been shown to inhibit or enhance soil OM decomposition dynamics, our study highlights that among all edaphic parameters, clay content best explains teabags’ degradation across sites. Therefore, future experiments that aim to manipulate soil mineral composition together with soil microbial communities and litter type could give more insights into how these different parameters interact to facilitate soil OM degradation.

### 4.3. The effect of microbial activity on litter decomposition

At the onset of this experiment, we predicted a general decrease in microbial functionality with increasing elevation, likely due to a rapid reduction in temperature-mediated enzymatic activity and loss of microbial biodiversity with elevation (Pellissier and Rasmann, 2018). Therefore, because microbial communities in the soil affect the carbon cycle through heterotrophic respiration (Bond-Lamberty et al., 2018), we expected that faster soil OM degradation in the subalpine versus the alpine zones, as a direct consequence of higher microbial activity in the subalpine zone. Accordingly, in our experiment, by using the EcoPlates’ bioassay, we observed that microbial carbon source consumption was higher in subalpine soils than in alpine soils. We here confirmed that microbial communities of mid-elevation can inherently degrade carbon sources faster than alpine microbial communities (Semeraro et al., 2022). Differences in soil microbial communities can be due to changes in the plant community structure and diversity (Cleveland et al., 2014), but also due to changing abiotic environmental conditions, such as temperature, humidity or solar radiations (Wei et al., 2022), or variation in soil physico-chemical properties, ultimately leading to different degradation potentials. Yet, these predictions were not reflected in the tea bag experiment. While we cannot exclude that changes in soil microbial community composition and activity, as was shown previously along the same transects (Semeraro et al., 2022), could also mediate variation in OM degradation, these effects were statistically dampened by the strong effect of solar radiation and other variable measured. Yet again, photodegradation alone could not explain OM degradation without the subsequent labor of the soil microbial communities. Thus, we suggest photodegradation facilitates the initial steps of OM degradation, which is then amplified by the bacterial and fungal community present at each site (Méndez et al., 2022).

General theory also indicates that soil microbial metabolic activity should mirror soil respiration, and therefore should increase with increasing temperatures (Johnston and Sibly, 2018). Accordingly, soil microbial communities, respiration, and temperature should be closely linked to litter decomposition processes (Joshi and Garkoti, 2020). Here, we found the opposite; alpine soils respired more than subalpine soils. Soil respiration, at least as we measured it, might also be the reflection of root respiration. Accordingly, since alpine meadows have a much higher root/soil volume ratio than forested subalpine habitats (Kergunteuil et al., 2016), our results of soil respiration were probably driven more by root-related processes than microbial activity. This observation might thus also explain why we did not detect a strong effect of soil microbial activity on teabags’ degradation.

## 5. Conclusion

This study highlights the role of both climatic and edaphic variables in decomposition processes, and highlights that variation in OM degradation is likely mediated by variation in photodegradation, as well as soil mineral composition, which can facilitate OM decomposition through the activity of soil microbes. We showed this by measuring the decomposition of two types of teas. While such a design allowed removing the effect of the local vegetation types, it has drawbacks: first, with this design we cannot extrapolate to how the local vegetation type – and phytochemistry therein – affects OM decomposition our experimental design. Second, we were not able to fully disentangle the effect of soil microbial activity from other ecological variables influencing OM degradation. Nonetheless, by highlighting a predominant role of climatic conditions (solar radiation) on OM decomposition, we suggest that these dynamics are likely to change with climate and temperature change. For instance, locally, higher photoperiods, in conjunction with increases in temperature, and inherent higher microbial activity, may cause faster decomposition rates, independently of exposition or elevation, ultimately spurring faster CO_2_ release in the atmosphere. Accordingly, future research manipulating solar radiation and its impacts on local soil microbial communities and decomposition should be conducted to address decomposition rates in the context of climate change.

## Data availability statement

The original contributions presented in the study are included in the article/Supplementary materials, further inquiries can be directed to the corresponding author.

## Author contributions

All authors contributed to conception and design of the study. SS and PK organized the database. SS and SR performed the statistical analysis. SS, PK, and SR wrote the first draft of the manuscript. All authors contributed to the article and approved the submitted version.

## Funding

This work was financed by the University of Neuchâtel, and by Swiss National Science foundation grants (31003A_179481 and 310030_204811) to SR.

## Conflict of interest

The authors declare that the research was conducted in the absence of any commercial or financial relationships that could be construed as a potential conflict of interest.

## Publisher’s note

All claims expressed in this article are solely those of the authors and do not necessarily represent those of their affiliated organizations, or those of the publisher, the editors and the reviewers. Any product that may be evaluated in this article, or claim that may be made by its manufacturer, is not guaranteed or endorsed by the publisher.
