# Supplementary material for: Solar radiation explains litter degradation along alpine elevation gradients better than other climatic or edaphic parameters
Source: Front Microbiol. 2023 Apr 27;14:1152187. doi: 10.3389/fmicb.2023.1152187 (PMC10174231; doi:10.3389/fmicb.2023.1152187)
Supplement: Supplementary file 1 [file Data_Sheet_1.PDF]

Supplementary Material

Solar radiation explains litter degradation along alpine elevation gradients better than other climatic or edaphic parameters

Sarah Semeraro*, Pascal Kipf, Claire Le Bayon, Sergio Rasmann

*** Correspondence:** Corresponding Author: sarah.semeraro@unine.ch

Table S1. Two-way ANOVA table for the effect of elevation zone (two levels; alpine and subalpine), exposition (two levels; north and south), region (two levels; Valais, Ticino), and interaction between zone and exposition on 11 soil physicochemical properties; total nitrogen (N_{tot}), total carbon (C_{tot}), carbon to nitrogen ratio (C/N), relative humidity (Rh), pH, soil organic matter (SOM), cation-exchange capacity (CEC), total carbonates ($CaCO_3$), clay, silt, and sand content.

Variable	Factor	Df	SSQ	F value	Pr (<F)	
Total nitrogen content (N_{tot})	Elevation Zone (Z)	1	0.0199	0.935	0.345711	
	Exposition (E)	1	0.0505	2.369	0.140275	
	Region	1	0.4056	19.033	0.000335	***
	Z * E	1	0.1337	6.273	0.021526	*
	Residuals	19	0.4049			
Total carbon content (C_{tot})	Elevation Zone (Z)	1	0.36	0.049	0.82662	
	Exposition (E)	1	25.32	3.423	0.07989	.
	Region	1	28.82	3.896	0.06312	.
	Z * E	1	79.92	10.805	0.00388	**
	Residuals	19	140.53			
Carbon-to-nitrogen ratio (CN)	Elevation Zone (Z)	1	2.6	0.305	0.587	
	Exposition (E)	1	1.9	0.216	0.647	
	Region	1	372.9	43.356	2.65e-06	***
	Z * E	1	6.1	0.712	0.409	
	Residuals	19	163.4			
Residual humidity (Rh)	Elevation Zone (Z)	1	10.31	2.263	0.149	
	Exposition (E)	1	2.93	0.643	0.432	
	Region	1	6.54	1.435	0.246	
	Z * E	1	31.17	6.837	0.017	*
	Residuals	19	86.61			
Soil organic matter (SOM)	Elevation Zone (Z)	1	4.51	1.106	0.30625	
	Exposition (E)	1	6.45	1.581	0.22391	
	Region	1	11.14	2.732	0.11480	
	Z * E	1	54.21	13.289	0.00172	**
	Residuals	19	77.50			
Soil water pH (pH)	Elevation Zone (Z)	1	0.004	0.006	0.93967	
	Exposition (E)	1	1.796	2.379	0.13947	
	Region	1	8.193	10.853	0.00381	**
	Z * E	1	0.548	0.726	0.40469	
	Residuals	19	14.342			
Soil cation-exchange-capacity (CEC)	Elevation Zone (Z)	1	388.1	5.787	0.027808	*
	Exposition (E)	1	448.3	6.685	0.019249	*
	Region	1	1101.3	16.421	0.000828	***
	Z * E	1	529.9	7.901	0.012027	*
	Residuals	17	1140.1			
Soil total carbonates content ($CaCO_3$)	Elevation Zone (Z)	1	0.1467	5.367	0.03326	*
	Exposition (E)	1	0.1598	5.845	0.02714	*
	Region	1	0.3961	14.492	0.00141	**
	Z * E	1	0.1607	5.879	0.02676	*
	Residuals	17	0.4647			
Soil clay content (clay)	Elevation Zone (Z)	1	3.6	0.276	0.605	
	Exposition (E)	1	8.7	0.662	0.426	
	Region	1	790.6	60.649	2.59e-07	***
	Z * E	1	1	0.074	0.788	
	Residuals	19	248.9			
Soil silt content (silt)	Elevation Zone (Z)	1	100	0.968	0.338	
	Exposition (E)	1	160	1.547	0.229	
	Region	1	4337	41.968	3.3e-06	***
	Z * E	1	16	0.156	0.698	
	Residuals	19	1964			
Soil sand content (sand)	Elevation Zone (Z)	1	467	5.809	0.0262	*
	Exposition (E)	1	639	7.961	0.0109	*
	Region	1	10748	133.823	4.8e-10	***
	Z * E	1	22	0.275	0.6059	
	Residuals	19	1526			

Table S2. Summary tables of all 19 measured variables used for the identification of solar radiation as the primary driver of both green and rooibos teabags decomposition rate.

Variable type	Variable	Measurement scale
Climate	Humidity	Raster layer of a climatic model (25m precision)
	Elevation	Raster layer of a climatic model (25m precision)
	Frostdays	Raster layer of a climatic model (25m precision)
	Precipitations	Raster layer of a climatic model (25m precision)
	Radiation	Raster layer of a climatic model (25m precision)
	Degreedays	Raster layer of a climatic model (25m precision)
Edaphic	Total nitrogen content (N_{tot})	Plot scale (400m^2 under forest and 25m^2 in grasslands)
	Total carbon content (C_{tot})	Plot scale (400m^2 under forest and 25m^2 in grasslands)
	Carbon-to-nitrogen ratio (CN)	Plot scale (400m^2 under forest and 25m^2 in grasslands)
	Residual humidity (Rh)	Plot scale (400m^2 under forest and 25m^2 in grasslands)
	Soil organic matter (SOM)	Plot scale (400m^2 under forest and 25m^2 in grasslands)
	Soil water pH (pH)	Plot scale (400m^2 under forest and 25m^2 in grasslands)
	Soil cation-exchange-capacity (CEC)	Plot scale (400m^2 under forest and 25m^2 in grasslands)
	Soil total carbonates content ($CaCO_3$)	Plot scale (400m^2 under forest and 25m^2 in grasslands)
	Soil clay content (clay)	Plot scale (400m^2 under forest and 25m^2 in grasslands)
	Soil silt content (silt)	Plot scale (400m^2 under forest and 25m^2 in grasslands)
	Soil sand content (sand)	Plot scale (400m^2 under forest and 25m^2 in grasslands)
Microbial	Soil respiration (CO_2 efflux)	Plot scale (n= 24 x 3 replicate measurements)
	Microbial carbon source consumption (EcoPlates)	Plot scale (n= 24 x 5 subsamples and 3 replicate measurements)

Table S3: Tea bag burial experiment. Details about the burial, the recollection date, and the respective exposure time in the field (days) are listed below for each plot.

Site	Burial	Recollection	Exposure time [days]
VNM (1 - 2 - 3)	30.9.2020	25.5.2021	237
VNH (1 - 2 - 3)	01.10.2020	30.6.2021	272
VSM (1 - 2 - 3)	30.9.2020	24.5.2021	236
VSH (1 - 2 - 3)	29.9.2020	30.6.2021	274
TNM (1 - 2 - 3)	09.10.2020	21.5.2021	224
TNH (1 - 2 - 3)	08.10.2020	29.6.2021	264
TSM (1 - 2 - 3	07.10.2020	21.5.2021	226
TSH (1 - 2 - 3)	07.10.2020	28.6.2021	264

Table S4: Soil respiration (LICOR) measurement settings.

Instrument name: 81A-0921	Software version 4.0.0	
Chamber size: d = 20 cm	Area = 317.8 cm2	V_{cham}ber = 4823.9 cm3 (autom).
V_{irga} = 19 cm3	Offset: adjusted specifically for each measurement position	
Pre-purge: 30 sec	Post-purge: 30 sec	Observation length: 120 sec

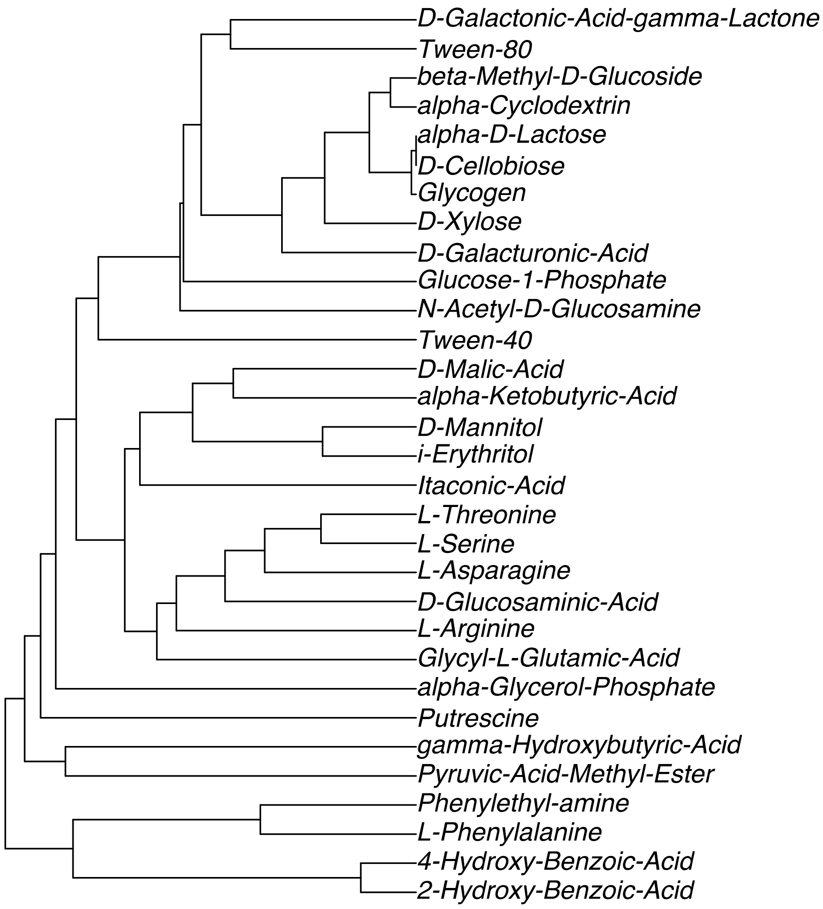

Figure S1. Chemical tree of all carbon sources in the EcoPlates.

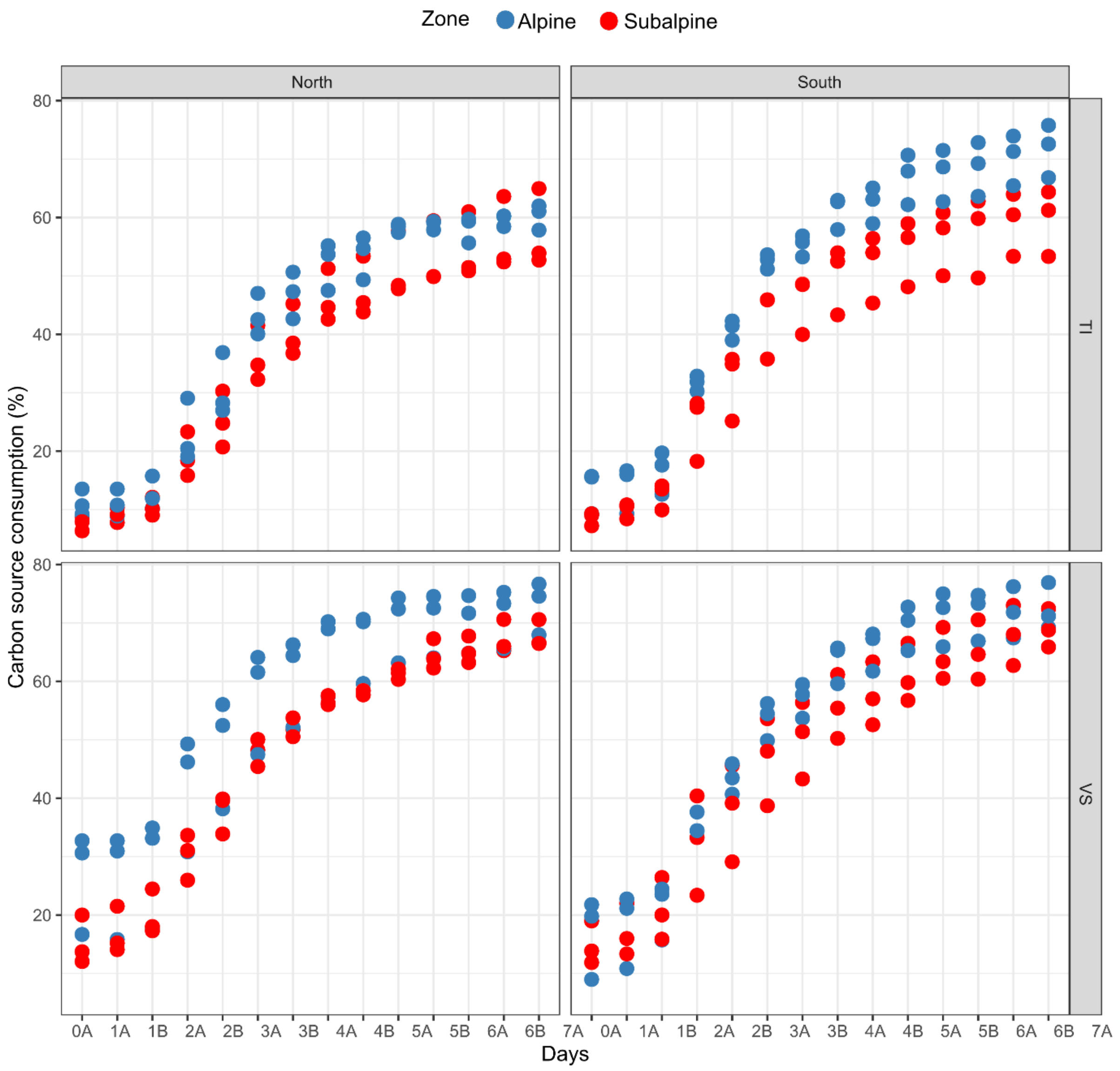

Figure S2. Carbon consumption across time and sites. Scatterplots show the evolution across seven days in the total carbon consumption (sum of all different carbon sources as shown in Figure S1) across the two regions (Ticino (TI) and Valais (VS), the north and south slopes, and in the subalpine (red dots) and alpine (blue dots) sites.

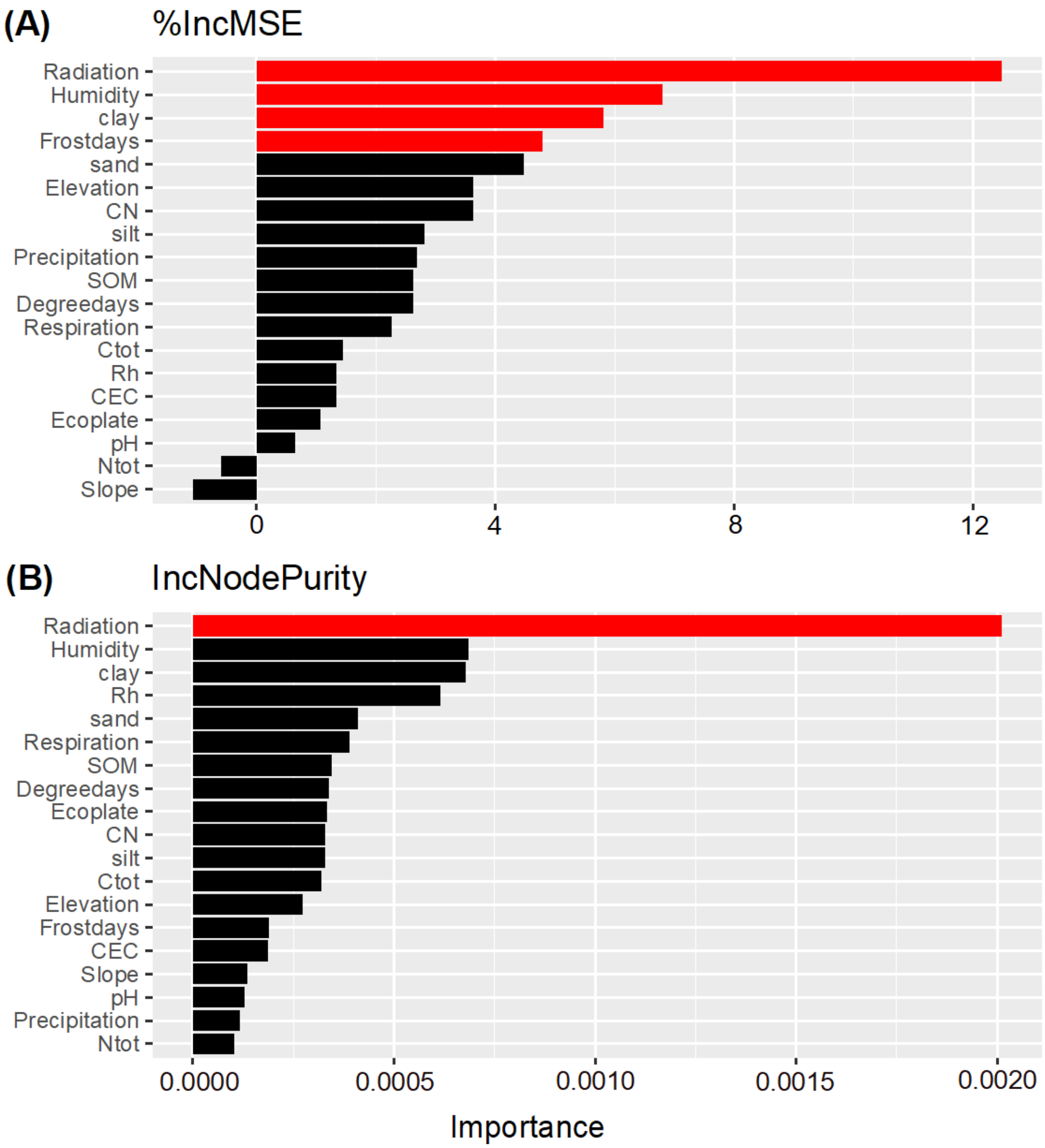

Figure S3. Random forest variable importance plot for the green tea degradation. Shown are (**A**) %IncMSE, indicating the average increase in squared residuals of the test set when variables are randomly permuted (little importance = little change in model when variable is removed or added), and (**B**) IncNodePurity, indicating the increase in homogeneity, for relating tea organic matter degradation to all climatic, edaphic and microbial-related variables. Red bars indicate significant values ($p < 0.05$) for explaining the importance of each response variable in the Random Forest model.

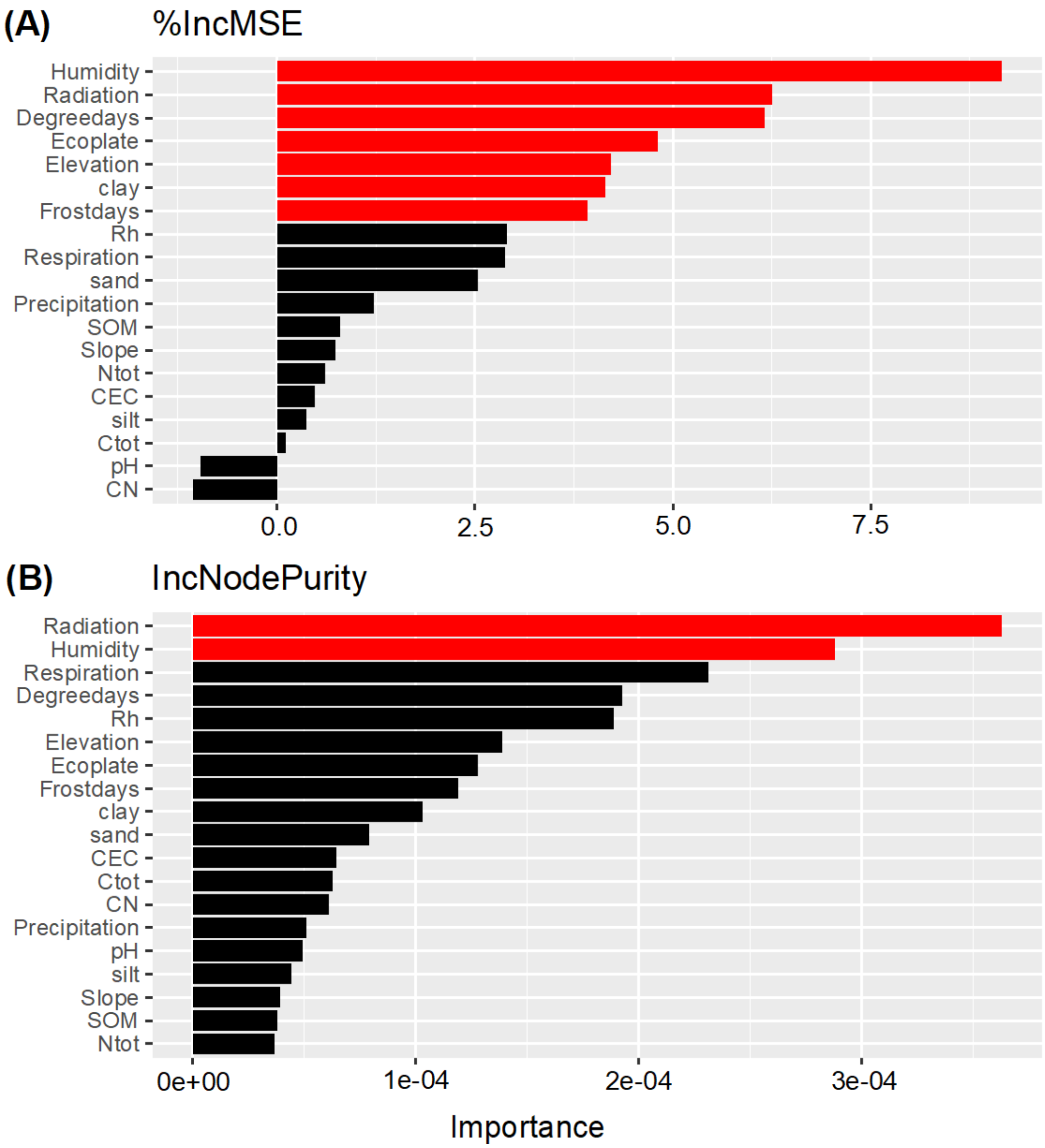

Figure S4. Random forest variable importance plot for the rooibos tea degradation. Shown are (**A**) %IncMSE, indicanting the average increase in squared residuals of the test set when variables are randomly permuted (little importance = little change in model when variable is removed or added), and (**B**) IncNodePurity, indicating the increase in homogeneity, for relating tea organic matter degradation to all climatic, edaphic and microbial-related variables. Red bars indicate significant values ($p < 0.05$) for explaining the importance of each response variable in the Random Forest model.

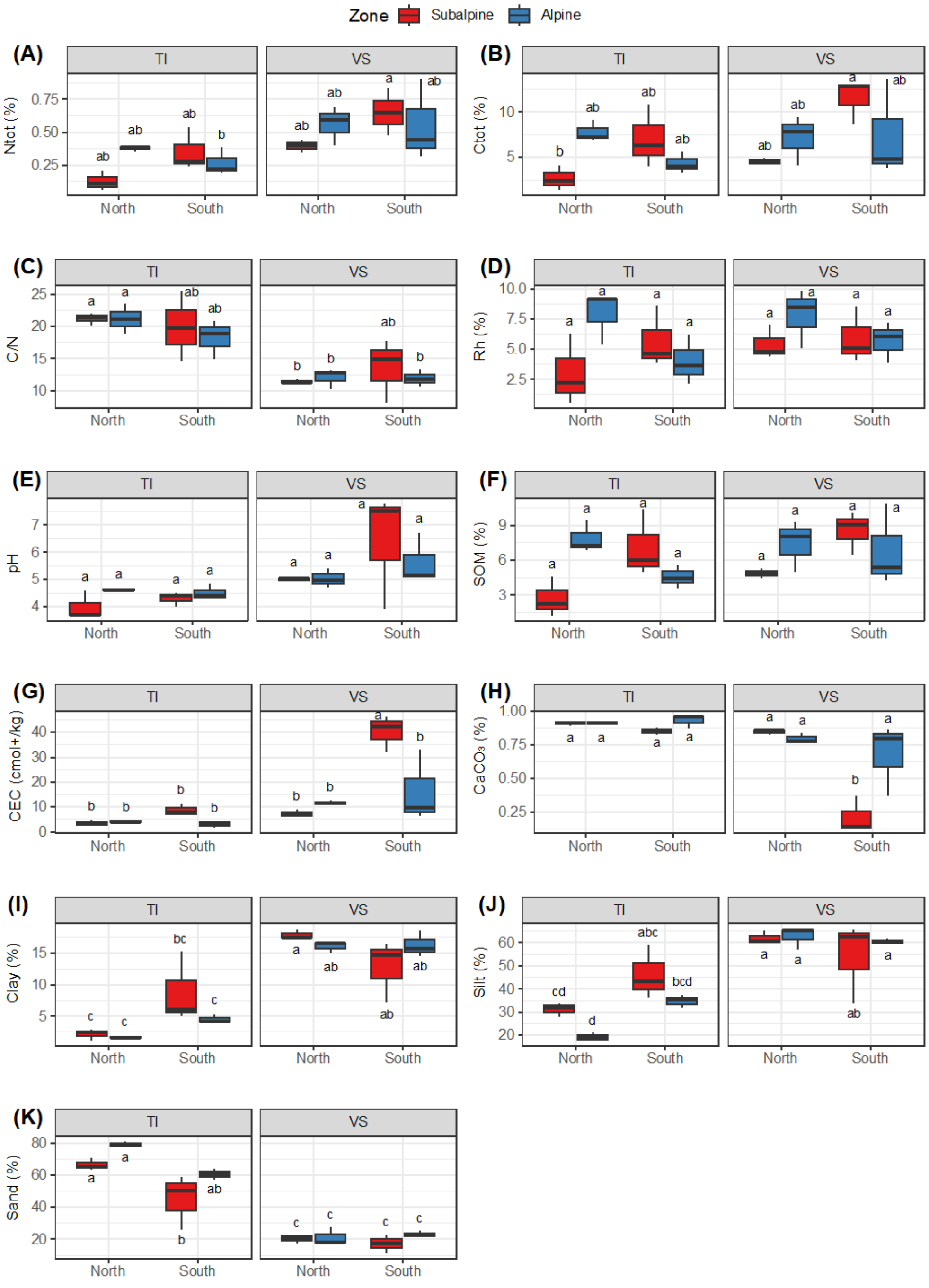

Figure S5. Soil physicochemical properties boxplot illustrating (**A**) soil total nitrogen content [N_{tot}], (**B**) soil total carbon content [C_{tot}], (**C**) the carbon-to-nitrogen ration [C/N], (**D**) soil residual humidity [RH], (**E**) soil water pH [pH], (**F**) soil organic matter content [SOM], (**G**) soil cation-exchange-capacity [CEC], (**H**) the total carbonate content [$CaCO_3$], (**I**) soil clay content [clay], (**J**) soil silt content [silt], (**K**) soil sand content [sand]. The boxplot shows differences in soil physicochemical properties across region, elevation and exposition. Colors indicate the two-elevation zone represented: alpine (blue), and subalpine sites (red).

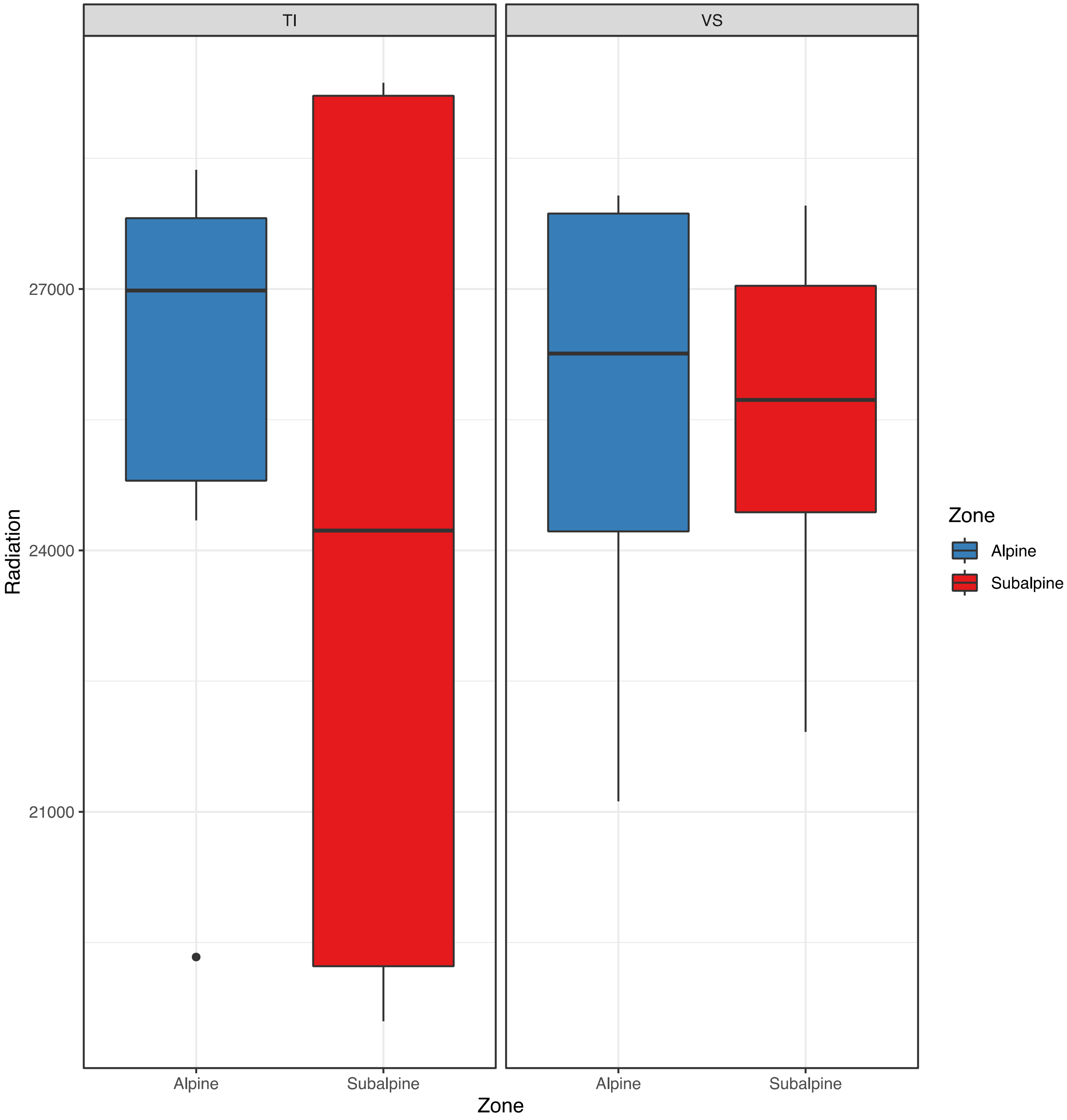

Figure S6. Effect of elevation on solar radiation. Boxplots representing average annual solar radiation across two regions (Ticino = TI, or Valais = VS) of the Swiss Alps. Red color (warm) represents the sub-alpine zone (~1400 m above sea level), and blue color (cold) represents the alpine zone (~2000 m above sea level). N = 24 sites. Solar radiation is independent of elevation (ANOVA; elevation effect; $F_{1,21} = 0.30$, $p = 0.59$, and region effect; $F_{1,21} = 0.19$, $p = 0.67$).
